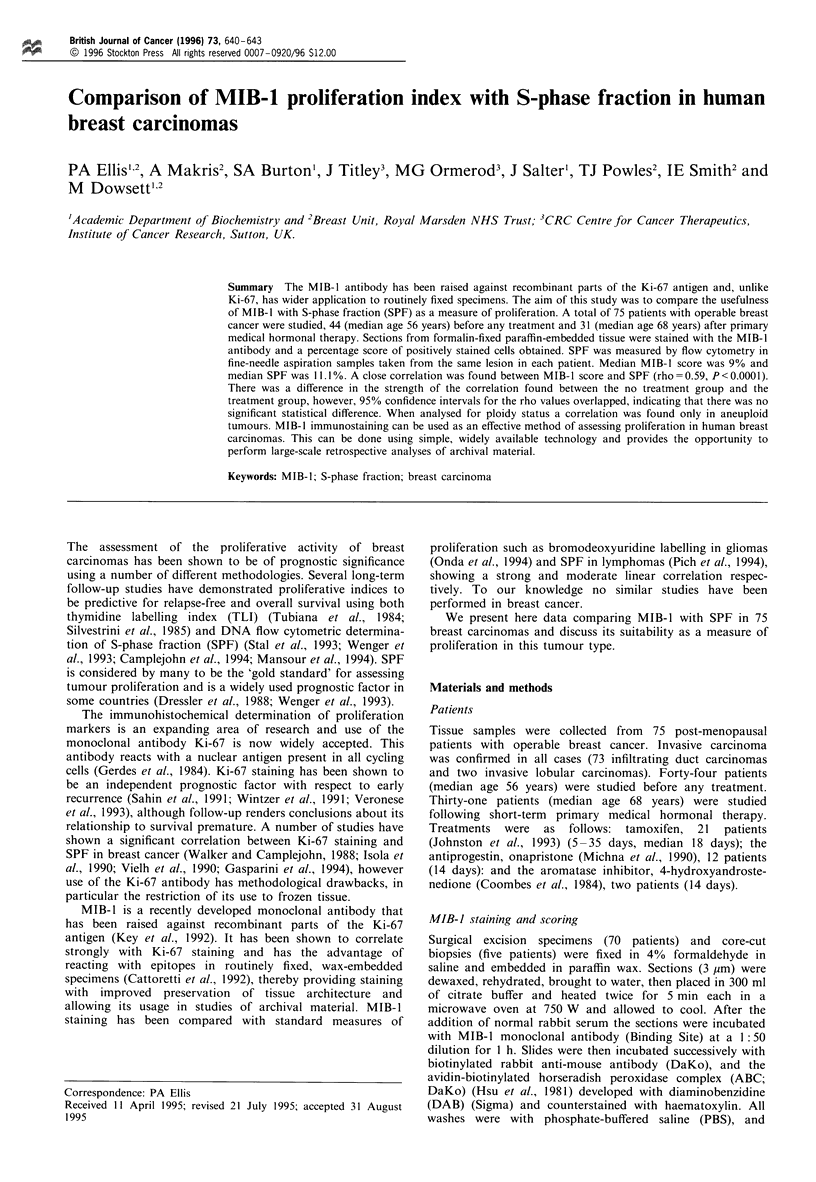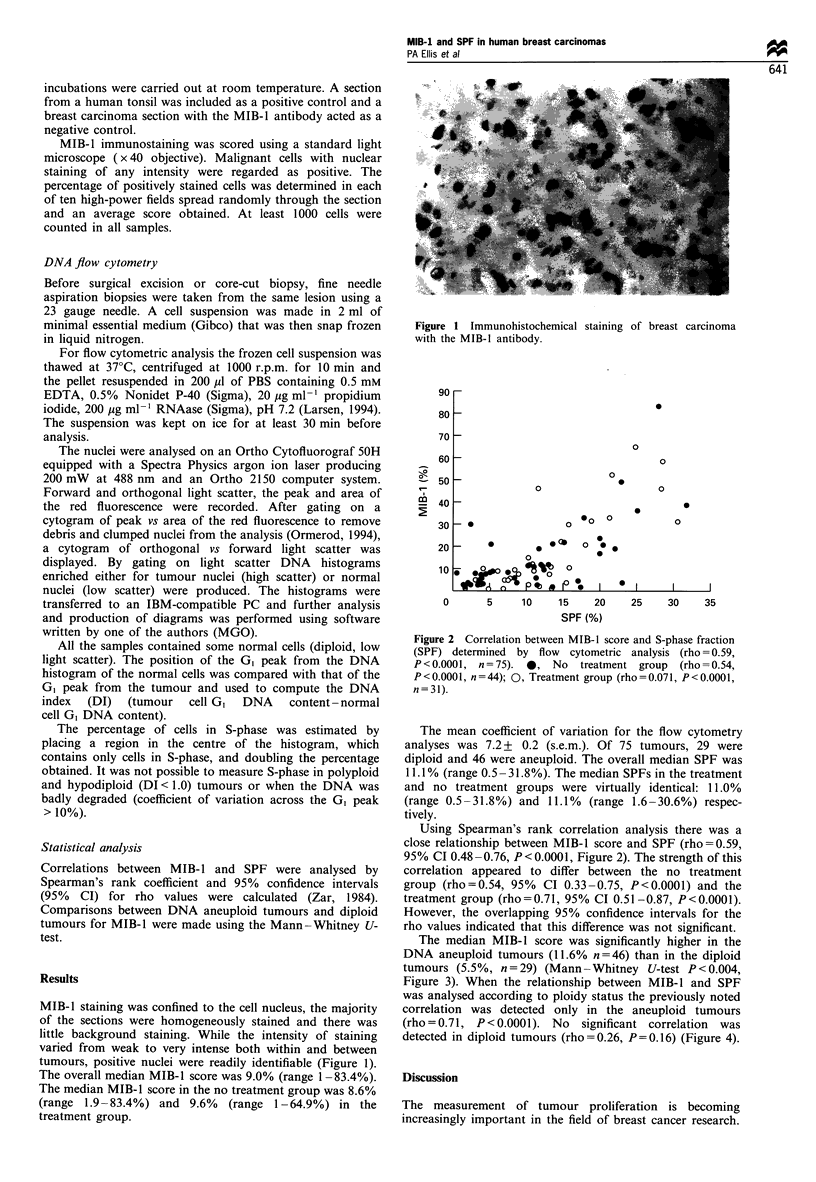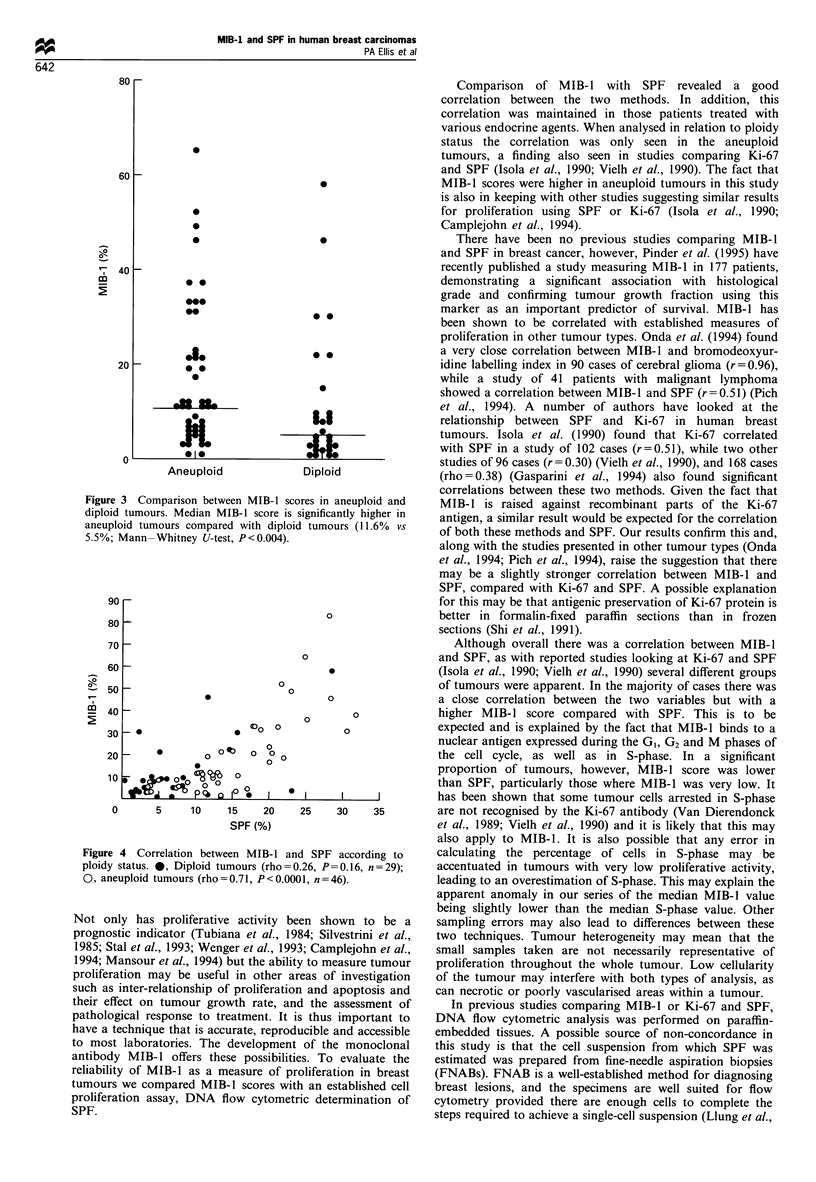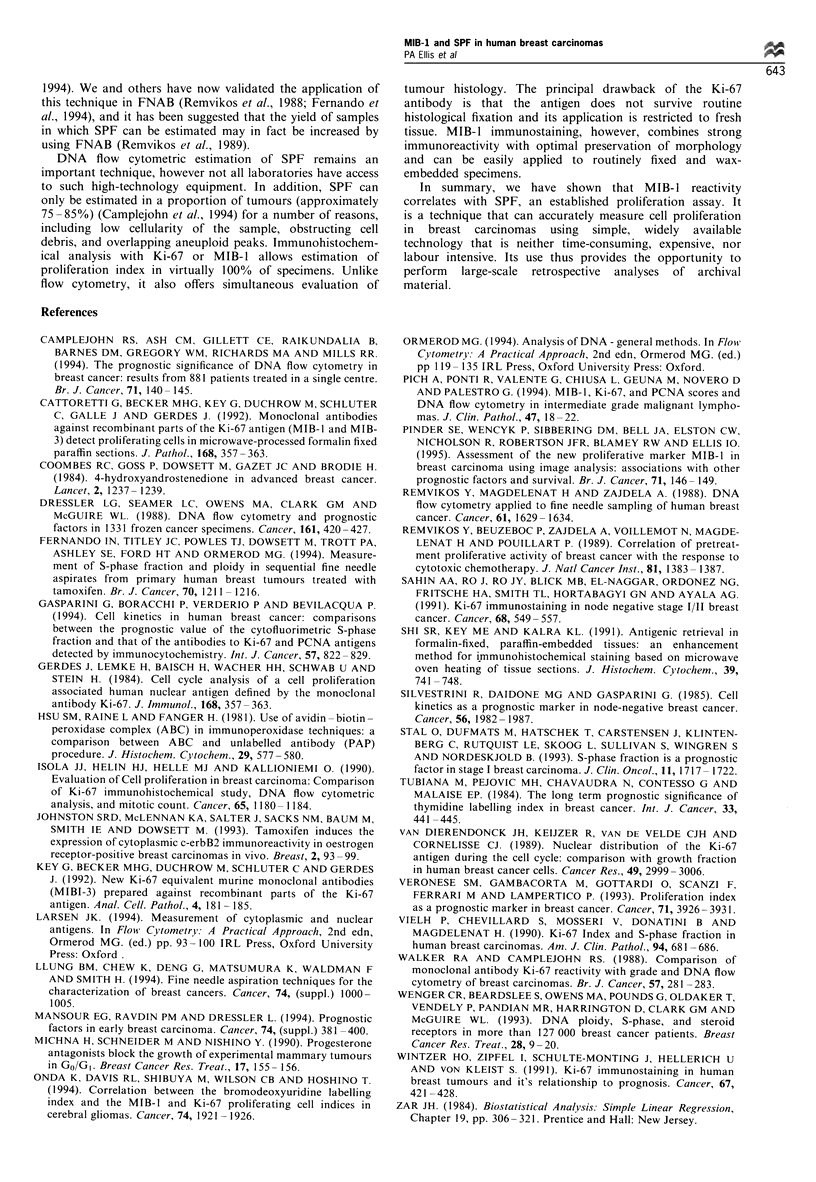# Comparison of MIB-1 proliferation index with S-phase fraction in human breast carcinomas.

**DOI:** 10.1038/bjc.1996.111

**Published:** 1996-03

**Authors:** P. A. Ellis, A. Makris, S. A. Burton, J. Titley, M. G. Ormerod, J. Salter, T. J. Powles, I. E. Smith, M. Dowsett

**Affiliations:** Academic Department of Biochemistry, Royal Marsden NHS Trust, Sutton, UK.

## Abstract

**Images:**


British Journal of Cancer (1996) 73, 640–643
© 1996 Stockton Press All rights reserved 0007-0920/96 $12.00

### Comparison of MIB-1 proliferation index with S-phase fraction in human breast carcinomas

PA Ellis[1,2], A Makris[2], SA Burton[1], J Titley[3], MG Ormerod[3], J Salter[1], TJ Powles[2], IE Smith[2] and M Dowsett[1,2]

[1]*Academic Department of Biochemistry and* [2]*Breast Unit, Royal Marsden NHS Trust;* [3]*CRC Centre for Cancer Therapeutics, Institute of Cancer Research, Sutton, UK.*

**Summary** The MIB-1 antibody has been raised against recombinant parts of the Ki-67 antigen and, unlike Ki-67, has wider application to routinely fixed specimens. The aim of this study was to compare the usefulness of MIB-1 with S-phase fraction (SPF) as a measure of proliferation. A total of 75 patients with operable breast cancer were studied, 44 (median age 56 years) before any treatment and 31 (median age 68 years) after primary medical hormonal therapy. Sections from formalin-fixed paraffin-embedded tissue were stained with the MIB-1 antibody and a percentage score of positively stained cells obtained. SPF was measured by flow cytometry in fine-needle aspiration samples taken from the same lesion in each patient. Median MIB-1 score was 9% and median SPF was 11.1%. A close correlation was found between MIB-1 score and SPF (rho = 0.59, $P < 0.0001$). There was a difference in the strength of the correlation found between the no treatment group and the treatment group, however, 95% confidence intervals for the rho values overlapped, indicating that there was no significant statistical difference. When analysed for ploidy status a correlation was found only in aneuploid tumours. MIB-1 immunostaining can be used as an effective method of assessing proliferation in human breast carcinomas. This can be done using simple, widely available technology and provides the opportunity to perform large-scale retrospective analyses of archival material.

**Keywords:** MIB-1; S-phase fraction; breast carcinoma

The assessment of the proliferative activity of breast carcinomas has been shown to be of prognostic significance using a number of different methodologies. Several long-term follow-up studies have demonstrated proliferative indices to be predictive for relapse-free and overall survival using both thymidine labelling index (TLI) (Tubiana *et al.*, 1984; Silvestrini *et al.*, 1985) and DNA flow cytometric determination of S-phase fraction (SPF) (Stal *et al.*, 1993; Wenger *et al.*, 1993; Camplejohn *et al.*, 1994; Mansour *et al.*, 1994). SPF is considered by many to be the 'gold standard' for assessing tumour proliferation and is a widely used prognostic factor in some countries (Dressler *et al.*, 1988; Wenger *et al.*, 1993).

The immunohistochemical determination of proliferation markers is an expanding area of research and use of the monoclonal antibody Ki-67 is now widely accepted. This antibody reacts with a nuclear antigen present in all cycling cells (Gerdes *et al.*, 1984). Ki-67 staining has been shown to be an independent prognostic factor with respect to early recurrence (Sahin *et al.*, 1991; Wintzer *et al.*, 1991; Veronese *et al.*, 1993), although follow-up renders conclusions about its relationship to survival premature. A number of studies have shown a significant correlation between Ki-67 staining and SPF in breast cancer (Walker and Camplejohn, 1988; Isola *et al.*, 1990; Vielh *et al.*, 1990; Gasparini *et al.*, 1994), however use of the Ki-67 antibody has methodological drawbacks, in particular the restriction of its use to frozen tissue.

MIB-1 is a recently developed monoclonal antibody that has been raised against recombinant parts of the Ki-67 antigen (Key *et al.*, 1992). It has been shown to correlate strongly with Ki-67 staining and has the advantage of reacting with epitopes in routinely fixed, wax-embedded specimens (Cattoretti *et al.*, 1992), thereby providing staining with improved preservation of tissue architecture and allowing its usage in studies of archival material. MIB-1 staining has been compared with standard measures of proliferation such as bromodeoxyuridine labelling in gliomas (Onda *et al.*, 1994) and SPF in lymphomas (Pich *et al.*, 1994), showing a strong and moderate linear correlation respectively. To our knowledge no similar studies have been performed in breast cancer.

We present here data comparing MIB-1 with SPF in 75 breast carcinomas and discuss its suitability as a measure of proliferation in this tumour type.

### Materials and methods

### *Patients*

Tissue samples were collected from 75 post-menopausal patients with operable breast cancer. Invasive carcinoma was confirmed in all cases (73 infiltrating duct carcinomas and two invasive lobular carcinomas). Forty-four patients (median age 56 years) were studied before any treatment. Thirty-one patients (median age 68 years) were studied following short-term primary medical hormonal therapy. Treatments were as follows: tamoxifen, 21 patients (Johnston *et al.*, 1993) (5–35 days, median 18 days); the antiprogestin, onapristone (Michna *et al.*, 1990), 12 patients (14 days): and the aromatase inhibitor, 4-hydroxyandrostenedione (Coombes *et al.*, 1984), two patients (14 days).

### *MIB-1 staining and scoring*

Surgical excision specimens (70 patients) and core-cut biopsies (five patients) were fixed in 4% formaldehyde in saline and embedded in paraffin wax. Sections (3 μm) were dewaxed, rehydrated, brought to water, then placed in 300 ml of citrate buffer and heated twice for 5 min each in a microwave oven at 750 W and allowed to cool. After the addition of normal rabbit serum the sections were incubated with MIB-1 monoclonal antibody (Binding Site) at a 1 : 50 dilution for 1 h. Slides were then incubated successively with biotinylated rabbit anti-mouse antibody (DaKo), and the avidin-biotinylated horseradish peroxidase complex (ABC; DaKo) (Hsu *et al.*, 1981) developed with diaminobenzidine (DAB) (Sigma) and counterstained with haematoxylin. All washes were with phosphate-buffered saline (PBS), and

Correspondence: PA Ellis
Received 11 April 1995; revised 21 July 1995; accepted 31 August 1995

incubations were carried out at room temperature. A section from a human tonsil was included as a positive control and a breast carcinoma section with the MIB-1 antibody acted as a negative control.

MIB-1 immunostaining was scored using a standard light microscope (×40 objective). Malignant cells with nuclear staining of any intensity were regarded as positive. The percentage of positively stained cells was determined in each of ten high-power fields spread randomly through the section and an average score obtained. At least 1000 cells were counted in all samples.

*DNA flow cytometry*

Before surgical excision or core-cut biopsy, fine needle aspiration biopsies were taken from the same lesion using a 23 gauge needle. A cell suspension was made in 2 ml of minimal essential medium (Gibco) that was then snap frozen in liquid nitrogen.

For flow cytometric analysis the frozen cell suspension was thawed at 37°C, centrifuged at 1000 r.p.m. for 10 min and the pellet resuspended in 200 $\mu$l of PBS containing 0.5 mM EDTA, 0.5% Nonidet P-40 (Sigma), 20 $\mu g\ ml^{-1}$ propidium iodide, 200 $\mu g\ ml^{-1}$ RNAase (Sigma), pH 7.2 (Larsen, 1994). The suspension was kept on ice for at least 30 min before analysis.

The nuclei were analysed on an Ortho Cytofluorograf 50H equipped with a Spectra Physics argon ion laser producing 200 mW at 488 nm and an Ortho 2150 computer system. Forward and orthogonal light scatter, the peak and area of the red fluorescence were recorded. After gating on a cytogram of peak *vs* area of the red fluorescence to remove debris and clumped nuclei from the analysis (Ormerod, 1994), a cytogram of orthogonal *vs* forward light scatter was displayed. By gating on light scatter DNA histograms enriched either for tumour nuclei (high scatter) or normal nuclei (low scatter) were produced. The histograms were transferred to an IBM-compatible PC and further analysis and production of diagrams was performed using software written by one of the authors (MGO).

All the samples contained some normal cells (diploid, low light scatter). The position of the $G_1$ peak from the DNA histogram of the normal cells was compared with that of the $G_1$ peak from the tumour and used to compute the DNA index (DI) (tumour cell $G_1$ DNA content–normal cell $G_1$ DNA content).

The percentage of cells in S-phase was estimated by placing a region in the centre of the histogram, which contains only cells in S-phase, and doubling the percentage obtained. It was not possible to measure S-phase in polyploid and hypodiploid (DI < 1.0) tumours or when the DNA was badly degraded (coefficient of variation across the $G_1$ peak > 10%).

*Statistical analysis*

Correlations between MIB-1 and SPF were analysed by Spearman's rank coefficient and 95% confidence intervals (95% CI) for rho values were calculated (Zar, 1984). Comparisons between DNA aneuploid tumours and diploid tumours for MIB-1 were made using the Mann–Whitney *U*-test.

### Results

MIB-1 staining was confined to the cell nucleus, the majority of the sections were homogeneously stained and there was little background staining. While the intensity of staining varied from weak to very intense both within and between tumours, positive nuclei were readily identifiable (Figure 1). The overall median MIB-1 score was 9.0% (range 1–83.4%). The median MIB-1 score in the no treatment group was 8.6% (range 1.9–83.4%) and 9.6% (range 1–64.9%) in the treatment group.

**Figure 1** Immunohistochemical staining of breast carcinoma with the MIB-1 antibody.

**Figure 2** Correlation between MIB-1 score and S-phase fraction (SPF) determined by flow cytometric analysis (rho = 0.59, $P < 0.0001$, $n = 75$). ●, No treatment group (rho = 0.54, $P < 0.0001$, $n = 44$); ○, Treatment group (rho = 0.071, $P < 0.0001$, $n = 31$).

The mean coefficient of variation for the flow cytometry analyses was 7.2± 0.2 (s.e.m.). Of 75 tumours, 29 were diploid and 46 were aneuploid. The overall median SPF was 11.1% (range 0.5–31.8%). The median SPFs in the treatment and no treatment groups were virtually identical: 11.0% (range 0.5–31.8%) and 11.1% (range 1.6–30.6%) respectively.

Using Spearman's rank correlation analysis there was a close relationship between MIB-1 score and SPF (rho = 0.59, 95% CI 0.48–0.76, $P < 0.0001$, Figure 2). The strength of this correlation appeared to differ between the no treatment group (rho = 0.54, 95% CI 0.33–0.75, $P < 0.0001$) and the treatment group (rho = 0.71, 95% CI 0.51–0.87, $P < 0.0001$). However, the overlapping 95% confidence intervals for the rho values indicated that this difference was not significant.

The median MIB-1 score was significantly higher in the DNA aneuploid tumours (11.6% $n = 46$) than in the diploid tumours (5.5%, $n = 29$) (Mann–Whitney *U*-test $P < 0.004$, Figure 3). When the relationship between MIB-1 and SPF was analysed according to ploidy status the previously noted correlation was detected only in the aneuploid tumours (rho = 0.71, $P < 0.0001$). No significant correlation was detected in diploid tumours (rho = 0.26, $P = 0.16$) (Figure 4).

### Discussion

The measurement of tumour proliferation is becoming increasingly important in the field of breast cancer research.

**Figure 3** Comparison between MIB-1 scores in aneuploid and diploid tumours. Median MIB-1 score is significantly higher in aneuploid tumours compared with diploid tumours (11.6% *vs* 5.5%; Mann–Whitney *U*-test, $P<0.004$).

**Figure 4** Correlation between MIB-1 and SPF according to ploidy status. ●, Diploid tumours (rho = 0.26, $P=0.16$, $n=29$); ○, aneuploid tumours (rho = 0.71, $P<0.0001$, $n=46$).

Not only has proliferative activity been shown to be a prognostic indicator (Tubiana *et al.*, 1984; Silvestrini *et al.*, 1985; Stal *et al.*, 1993; Wenger *et al.*, 1993; Camplejohn *et al.*, 1994; Mansour *et al.*, 1994) but the ability to measure tumour proliferation may be useful in other areas of investigation such as inter-relationship of proliferation and apoptosis and their effect on tumour growth rate, and the assessment of pathological response to treatment. It is thus important to have a technique that is accurate, reproducible and accessible to most laboratories. The development of the monoclonal antibody MIB-1 offers these possibilities. To evaluate the reliability of MIB-1 as a measure of proliferation in breast tumours we compared MIB-1 scores with an established cell proliferation assay, DNA flow cytometric determination of SPF.

Comparison of MIB-1 with SPF revealed a good correlation between the two methods. In addition, this correlation was maintained in those patients treated with various endocrine agents. When analysed in relation to ploidy status the correlation was only seen in the aneuploid tumours, a finding also seen in studies comparing Ki-67 and SPF (Isola *et al.*, 1990; Vielh *et al.*, 1990). The fact that MIB-1 scores were higher in aneuploid tumours in this study is also in keeping with other studies suggesting similar results for proliferation using SPF or Ki-67 (Isola *et al.*, 1990; Camplejohn *et al.*, 1994).

There have been no previous studies comparing MIB-1 and SPF in breast cancer, however, Pinder *et al.* (1995) have recently published a study measuring MIB-1 in 177 patients, demonstrating a significant association with histological grade and confirming tumour growth fraction using this marker as an important predictor of survival. MIB-1 has been shown to be correlated with established measures of proliferation in other tumour types. Onda *et al.* (1994) found a very close correlation between MIB-1 and bromodeoxyuridine labelling index in 90 cases of cerebral glioma ($r=0.96$), while a study of 41 patients with malignant lymphoma showed a correlation between MIB-1 and SPF ($r=0.51$) (Pich *et al.*, 1994). A number of authors have looked at the relationship between SPF and Ki-67 in human breast tumours. Isola *et al.* (1990) found that Ki-67 correlated with SPF in a study of 102 cases ($r=0.51$), while two other studies of 96 cases ($r=0.30$) (Vielh *et al.*, 1990), and 168 cases (rho = 0.38) (Gasparini *et al.*, 1994) also found significant correlations between these two methods. Given the fact that MIB-1 is raised against recombinant parts of the Ki-67 antigen, a similar result would be expected for the correlation of both these methods and SPF. Our results confirm this and, along with the studies presented in other tumour types (Onda *et al.*, 1994; Pich *et al.*, 1994), raise the suggestion that there may be a slightly stronger correlation between MIB-1 and SPF, compared with Ki-67 and SPF. A possible explanation for this may be that antigenic preservation of Ki-67 protein is better in formalin-fixed paraffin sections than in frozen sections (Shi *et al.*, 1991).

Although overall there was a correlation between MIB-1 and SPF, as with reported studies looking at Ki-67 and SPF (Isola *et al.*, 1990; Vielh *et al.*, 1990) several different groups of tumours were apparent. In the majority of cases there was a close correlation between the two variables but with a higher MIB-1 score compared with SPF. This is to be expected and is explained by the fact that MIB-1 binds to a nuclear antigen expressed during the $G_1$, $G_2$ and M phases of the cell cycle, as well as in S-phase. In a significant proportion of tumours, however, MIB-1 score was lower than SPF, particularly those where MIB-1 was very low. It has been shown that some tumour cells arrested in S-phase are not recognised by the Ki-67 antibody (Van Dierendonck *et al.*, 1989; Vielh *et al.*, 1990) and it is likely that this may also apply to MIB-1. It is also possible that any error in calculating the percentage of cells in S-phase may be accentuated in tumours with very low proliferative activity, leading to an overestimation of S-phase. This may explain the apparent anomaly in our series of the median MIB-1 value being slightly lower than the median S-phase value. Other sampling errors may also lead to differences between these two techniques. Tumour heterogeneity may mean that the small samples taken are not necessarily representative of proliferation throughout the whole tumour. Low cellularity of the tumour may interfere with both types of analysis, as can necrotic or poorly vascularised areas within a tumour.

In previous studies comparing MIB-1 or Ki-67 and SPF, DNA flow cytometric analysis was performed on paraffin-embedded tissues. A possible source of non-concordance in this study is that the cell suspension from which SPF was estimated was prepared from fine-needle aspiration biopsies (FNABs). FNAB is a well-established method for diagnosing breast lesions, and the specimens are well suited for flow cytometry provided there are enough cells to complete the steps required to achieve a single-cell suspension (Llung *et al.*,

1994). We and others have now validated the application of this technique in FNAB (Remvikos *et al*., 1988; Fernando *et al*., 1994), and it has been suggested that the yield of samples in which SPF can be estimated may in fact be increased by using FNAB (Remvikos *et al*., 1989).

DNA flow cytometric estimation of SPF remains an important technique, however not all laboratories have access to such high-technology equipment. In addition, SPF can only be estimated in a proportion of tumours (approximately 75–85%) (Camplejohn *et al*., 1994) for a number of reasons, including low cellularity of the sample, obstructing cell debris, and overlapping aneuploid peaks. Immunohistochemical analysis with Ki-67 or MIB-1 allows estimation of proliferation index in virtually 100% of specimens. Unlike flow cytometry, it also offers simultaneous evaluation of tumour histology. The principal drawback of the Ki-67 antibody is that the antigen does not survive routine histological fixation and its application is restricted to fresh tissue. MIB-1 immunostaining, however, combines strong immunoreactivity with optimal preservation of morphology and can be easily applied to routinely fixed and wax-embedded specimens.

In summary, we have shown that MIB-1 reactivity correlates with SPF, an established proliferation assay. It is a technique that can accurately measure cell proliferation in breast carcinomas using simple, widely available technology that is neither time-consuming, expensive, nor labour intensive. Its use thus provides the opportunity to perform large-scale retrospective analyses of archival material.

### References

CAMPLEJOHN RS, ASH CM, GILLETT CE, RAIKUNDALIA B, BARNES DM, GREGORY WM, RICHARDS MA AND MILLS RR. (1994). The prognostic significance of DNA flow cytometry in breast cancer: results from 881 patients treated in a single centre. *Br. J. Cancer*, **71,** 140–145.

CATTORETTI G, BECKER MHG, KEY G, DUCHROW M, SCHLUTER C, GALLE J AND GERDES J. (1992). Monoclonal antibodies against recombinant parts of the Ki-67 antigen (MIB-1 and MIB-3) detect proliferating cells in microwave-processed formalin fixed paraffin sections. *J. Pathol*., **168,** 357–363.

COOMBES RC, GOSS P, DOWSETT M, GAZET JC AND BRODIE H. (1984). 4-hydroxyandrostenedione in advanced breast cancer. *Lancet*, **2,** 1237–1239.

DRESSLER LG, SEAMER LC, OWENS MA, CLARK GM AND McGUIRE WL. (1988). DNA flow cytometry and prognostic factors in 1331 frozen cancer specimens. *Cancer*, **161,** 420–427.

FERNANDO IN, TITLEY JC, POWLES TJ, DOWSETT M, TROTT PA, ASHLEY SE, FORD HT AND ORMEROD MG. (1994). Measurement of S-phase fraction and ploidy in sequential fine needle aspirates from primary human breast tumours treated with tamoxifen. *Br. J. Cancer*, **70,** 1211–1216.

GASPARINI G, BORACCHI P, VERDERIO P AND BEVILACQUA P. (1994). Cell kinetics in human breast cancer: comparisons between the prognostic value of the cytofluorimetric S-phase fraction and that of the antibodies to Ki-67 and PCNA antigens detected by immunocytochemistry. *Int. J. Cancer*, **57,** 822–829.

GERDES J, LEMKE H, BAISCH H, WACHER HH, SCHWAB U AND STEIN H. (1984). Cell cycle analysis of a cell proliferation associated human nuclear antigen defined by the monoclonal antibody Ki-67. *J. Immunol*., **168,** 357–363.

HSU SM, RAINE L AND FANGER H. (1981). Use of avidin–biotin–peroxidase complex (ABC) in immunoperoxidase techniques: a comparison between ABC and unlabelled antibody (PAP) procedure. *J. Histochem. Cytochem*., **29,** 577–580.

ISOLA JJ, HELIN HJ, HELLE MJ AND KALLIONIEMI O. (1990). Evaluation of Cell proliferation in breast carcinoma: Comparison of Ki-67 immunohistochemical study, DNA flow cytometric analysis, and mitotic count. *Cancer*, **65,** 1180–1184.

JOHNSTON SRD, McLENNAN KA, SALTER J, SACKS NM, BAUM M, SMITH IE AND DOWSETT M. (1993). Tamoxifen induces the expression of cytoplasmic c-erbB2 immunoreactivity in oestrogen receptor-positive breast carcinomas in vivo. *Breast*, **2,** 93–99.

KEY G, BECKER MHG, DUCHROW M, SCHLUTER C AND GERDES J. (1992). New Ki-67 equivalent murine monoclonal antibodies (MIBI-3) prepared against recombinant parts of the Ki-67 antigen. *Anal. Cell. Pathol*., **4,** 181–185.

LARSEN JK. (1994). Measurement of cytoplasmic and nuclear antigens. In *Flow Cytometry: A Practical Approach*, 2nd edn, Ormerod MG. (ed.) pp. 93–100 IRL Press, Oxford University Press: Oxford .

LLUNG BM, CHEW K, DENG G, MATSUMURA K, WALDMAN F AND SMITH H. (1994). Fine needle aspiration techniques for the characterization of breast cancers. *Cancer*, **74,** (suppl.) 1000–1005.

MANSOUR EG, RAVDIN PM AND DRESSLER L. (1994). Prognostic factors in early breast carcinoma. *Cancer*, **74,** (suppl.) 381–400.

MICHNA H, SCHNEIDER M AND NISHINO Y. (1990). Progesterone antagonists block the growth of experimental mammary tumours in $G_0/G_1$. *Breast Cancer Res. Treat*., **17,** 155–156.

ONDA K, DAVIS RL, SHIBUYA M, WILSON CB AND HOSHINO T. (1994). Correlation between the bromodeoxyuridine labelling index and the MIB-1 and Ki-67 proliferating cell indices in cerebral gliomas. *Cancer*, **74,** 1921–1926.

ORMEROD MG. (1994). Analysis of DNA - general methods. In *Flow Cytometry: A Practical Approach*, 2nd edn, Ormerod MG. (ed.) pp 119–135 IRL Press, Oxford University Press: Oxford.

PICH A, PONTI R, VALENTE G, CHIUSA L, GEUNA M, NOVERO D AND PALESTRO G. (1994). MIB-1, Ki-67, and PCNA scores and DNA flow cytometry in intermediate grade malignant lymphomas. *J. Clin. Pathol*., **47,** 18–22.

PINDER SE, WENCYK P, SIBBERING DM, BELL JA, ELSTON CW, NICHOLSON R, ROBERTSON JFR, BLAMEY RW AND ELLIS IO. (1995). Assessment of the new proliferative marker MIB-1 in breast carcinoma using image analysis: associations with other prognostic factors and survival. *Br. J. Cancer*, **71,** 146–149.

REMVIKOS Y, MAGDELENAT H AND ZAJDELA A. (1988). DNA flow cytometry applied to fine needle sampling of human breast cancer. *Cancer*, **61,** 1629–1634.

REMVIKOS Y, BEUZEBOC P, ZAJDELA A, VOILLEMOT N, MAGDELENAT H AND POUILLART P. (1989). Correlation of pretreatment proliferative activity of breast cancer with the response to cytotoxic chemotherapy. *J. Natl Cancer Inst*., **81,** 1383–1387.

SAHIN AA, RO J, RO JY, BLICK MB, EL-NAGGAR, ORDONEZ NG, FRITSCHE HA, SMITH TL, HORTABAGYI GN AND AYALA AG. (1991). Ki-67 immunostaining in node negative stage I/II breast cancer. *Cancer*, **68,** 549–557.

SHI SR, KEY ME AND KALRA KL. (1991). Antigenic retrieval in formalin-fixed, paraffin-embedded tissues: an enhancement method for immunohistochemical staining based on microwave oven heating of tissue sections. *J. Histochem. Cytochem*., **39,** 741–748.

SILVESTRINI R, DAIDONE MG AND GASPARINI G. (1985). Cell kinetics as a prognostic marker in node-negative breast cancer. *Cancer*, **56,** 1982–1987.

STAL O, DUFMATS M, HATSCHEK T, CARSTENSEN J, KLINTENBERG C, RUTQUIST LE, SKOOG L, SULLIVAN S, WINGREN S AND NORDESKJOLD B. (1993). S-phase fraction is a prognostic factor in stage I breast carcinoma. *J. Clin. Oncol*., **11,** 1717–1722.

TUBIANA M, PEJOVIC MH, CHAVAUDRA N, CONTESSO G AND MALAISE EP. (1984). The long term prognostic significance of thymidine labelling index in breast cancer. *Int. J. Cancer*, **33,** 441–445.

VAN DIERENDONCK JH, KEIJZER R, VAN DE VELDE CJH AND CORNELISSE CJ. (1989). Nuclear distribution of the Ki-67 antigen during the cell cycle: comparison with growth fraction in human breast cancer cells. *Cancer Res*., **49,** 2999–3006.

VERONESE SM, GAMBACORTA M, GOTTARDI O, SCANZI F, FERRARI M AND LAMPERTICO P. (1993). Proliferation index as a prognostic marker in breast cancer. *Cancer*, **71,** 3926–3931.

VIELH P, CHEVILLARD S, MOSSERI V, DONATINI B AND MAGDELENAT H. (1990). Ki-67 Index and S-phase fraction in human breast carcinomas. *Am. J. Clin. Pathol*., **94,** 681–686.

WALKER RA AND CAMPLEJOHN RS. (1988). Comparison of monoclonal antibody Ki-67 reactivity with grade and DNA flow cytometry of breast carcinomas. *Br. J. Cancer*, **57,** 281–283.

WENGER CR, BEARDSLEE S, OWENS MA, POUNDS G, OLDAKER T, VENDELY P, PANDIAN MR, HARRINGTON D, CLARK GM AND McGUIRE WL. (1993). DNA ploidy, S-phase, and steroid receptors in more than 127 000 breast cancer patients. *Breast Cancer Res. Treat*., **28,** 9–20.

WINTZER HO, ZIPFEL I, SCHULTE-MONTING J, HELLERICH U AND VON KLEIST S. (1991). Ki-67 immunostaining in human breast tumours and it's relationship to prognosis. *Cancer*, **67,** 421–428.

ZAR JH. (1984). *Biostatistical Analysis: Simple Linear Regression*, Chapter 19, pp. 306–321. Prentice and Hall: New Jersey.